# Effect of OLIG1 on the development of oligodendrocytes and myelination in a neonatal rat PVL model induced by hypoxia-ischemia

**DOI:** 10.3892/mmr.2014.3028

**Published:** 2014-12-01

**Authors:** TONGFEI CHENG, XINDONG XUE, JIANHUA FU

**Affiliations:** Department of Pediatrics, Shengjing Hospital of China Medical University, Shenyang, Liaoning 110004, P.R. China

**Keywords:** OLIG1, PVL, oligodendrocyte, myelin, hypoxia-ischemia, neonatal

## Abstract

OLIG1 is an oligodendrocyte (OL) transcription factor, which can contribute to the proliferation and differentiation of OLs, and the maturation of myelin. The aim of this study was to clarify the role of OLIG1 in neonatal Sprague Dawley rats with periventricular leukomalacia (PVL), induced by hypoxia-ischemia (HI). Newborn rats in the HI group were subjected to ligation of the right carotid artery, followed by 8% oxygen delivery for 2 h, while rats in the normoxia group were only subjected to isolation of the right carotid artery, without exposure to hypoxia. Samples of brain tissue from rats in both groups were collected at 1, 3, 7, 14 and 21 days. In the HI group, observation by transmission electron microscopy (TEM) revealed OLs with a damaged nuclear membrane, cellular atrophy, deformation and necrosis, and cells in myelin with a high number of small vacuoles. A double-label immunofluorescence assay revealed the translocation of OLIG1 from the cytoplasm to the nucleus, while western blot and reverse transcription-quantitative polymerase chain reaction assays showed that there is a significant decrease, followed by an increase, in the gene and protein expression levels of OLIG1 and myelin basic protein (MBP). Despite the increase at the late stages of HI, the final levels of these proteins remained lower than the corresponding levels in the normoxia group. In conclusion, the decreased protein expression of OLIG1 following HI plays an important role in inhibiting the development and maturation of OLs and myelin. Although OLIG1 may, via its nuclear translocation, promote the growth and development of myelin to a certain extent, this factor fails to fully repair injured myelin.

## Introduction

White matter damage (WMD) is the most common manifestation of brain injury in premature infants. Periventricular leukomalacia (PVL), as the most serious manifestation of WMD, influences the development and maturation of myelin, and may also result in severe neurologic deficits. The etiology of PVL is complex, but the major cause is hypoxia-ischemia (HI), which is also a primary contributor to brain injury in premature infants (1). No effective agent for the prevention and treatment of PVL has been developed, and the pathogenesis of PVL remains unclear. Thus, there is an urgent need to further study the pathogenesis of PVL, with the aim of identifying new targets for its treatment and prevention.

PVL is pathologically characterized by diffuse injury of the oligodendrocytes (OLs) and dysmyelination (2,3). Oligodendrocytes contribute to the myelination of the central nervous system, and have received much attention regarding their role in PVL-related injuries. Current studies on the role of PVL have focused on the abnormal secretion of inflammatory factors, the damage produced by oxyradicals, and hemodynamics (4,5), but interpretation of the results of these studies has been less than satisfactory.

The expression of the OL transcription factor OLIG1 was detected in immature oligodendrocyte precursor cells (OPCs) of the nervous system (6). OLIG1 can promote not only the differentiation of nerve stem cells (NSCs) to OLs (7), but also, the development and maturation of OLs and myelin (8,9). However, the association between OLIG1 and OLs or myelin in association with PVL has not been well established. This study aimed to further explore the effects of OLIG1 on OLs and myelin, which are affected by the processes occuring during PVL.

Previous studies have shown that OLIG1 not only regulates myelin, but is also involved in the repair of injured myelin (10,11). Xin *et al* (10) reported that OLIG1 is present in the nuclei of rat OPCs at the embryonic stage, and then translocates to the cytoplasm after birth. However, OLIG1 translocates back into the nuclei of OPCs following a demyelinating injury. Nuclear OLIG1 also plays an important role in the activation of other transcription factors, such as Sox10, Sox9 and OLIG2 (12). Furthermore, an association between OLIG1 expression and certain disorders of the central nervous system, e.g., the repair of myelin in multiple sclerosis, was previously reported (11); however, it is still unknown whether OLIG1 is associated with PVL in premature infants. An explanation for the important role of the nuclear translocation of OLIG1 in the repair and regeneration of myelin following HI-induced PVL would be highly valuable, for both the prevention and the development of new therapies for PVL. In this study, the effects of OLIG1 on PVL-affected OLs and myelin are discussed in detail.

## Materials and methods

### Animals and experimental groups

Eighty rats (3 days-old) of either gender, spontaneously delivered by pregnant rats on days 21–23 of pregnancy, were randomly divided into the HI group (n=40) and the normoxia group (n=40). The animals were provided by the Animal Department Experiment Center, of the Shengjing Hospital of China Medical University. The present study was approved by the Ethical Committee of China Medical University (Shenyang, China). An HI-induced animal model for PVL was established for the HI group according to the method reported by Mizuno *et al* (13). Ligation of the right common carotid artey was then performed following inhalation anesthesia, in order to reduce the pain. Rats in the HI group were then subjected to 2 h of exposure to hypoxic conditions (using a mixture of 8% O_2_ and 92% N_2_), while rats in the normoxia group were only subjected to isolation of their right common carotid artery. Samples of brain tissue were collected at 1, 3, 7, 14 and 21 days after HI exposure.

### Hematoxylin and eosin (H&E) staining

Samples of brain tissues were resected at locations within ±5 mm of the optic chiasma, embedded in paraffin blocks, cut into continuous coronal sections of 5 μm thickness, and finally stained with H&E (Beijing Zhongshan Goldenbridge Biotechnology, Beijing,China). Six sections performed at each time-point were randomly selected from each group, and then, the periventricular alba was observed for pathological changes in 5 random fields of a light microscope (magnification, ×400; Olympus Corporation, Tokyo, Japan).

### Transmission electron microscope (TEM)

The brain tissues were initially fixed in 2.5% glutaral at 4°C for 24 h, then treated in 1% osmic acid, dehydrated with acetone, and embedded in epoxy resin. The embedded tissues were cut into ultrathin sections and double-stained with uranyl acetate and lead nitrate. The ultrastructure of OLs and myelin was observed using a JEOL JEM-1200EX TEM (magnification, ×25,000; Hitachi High Technologies Corp., Tokyo, Japan).

### Immunohistochemistry

Sections of brain tissue were deparaffinized in graded alcohol solutions and xylene. The sections were then blocked with 3% H_2_O_2_ (37°C, 30 min) and goat serum (37°C, 20 min), followed by addition of rabbit anti-rat monoclonal anti-OLIG1 (1:300 dilution; Abcam, San Francisco, CA, USA) and incubation overnight at 4°C. The negative control tissues were incubated with phosphate-buffered saline (PBS) instead of the primary antibody, and also incubated with the biotin-labeled goat anti-rabbit IgG secondary antibody (37°C, 30 min), similarly to the tissues of interest. All sections were then incubated with a horseradish peroxidase (HRP) marker (37°C, 30 min), stained with 3′-diaminobenzidine (DAB), re-stained with hematoxylin, dehydrated, vitrified, and mounted. Ten different sections from each time-point were randomly selected from each group, and the periventricular alba was observed in 5 random fields of a light microscope (magnification, ×400).

### Immunofluorescence staining

Sections of brain tissue were infiltrated with 4% paraformaldehyde, soaked in 3% paraformaldehyde for 3 h at 4°C, cryoprotected in 30% sucrose for 12 h at 4°C, and frozen at 80°C. The frozen sections (10 μM thick) were air-dried and washed 3 times with PBS, incubated with 0.5% Triton X-100 for 5 min at room temperature, and again washed 3 times with PBS. The sections were then blocked with 10% goat serum for 30 min at 37°C, and incubated with the mouse monoclonal primary antibodies anti-oligodendrocyte marker O4 and anti-OLIG1 (both at 1:100 dilution; R&D Systems, Minneapolis, MN, USA) overnight at 4°C. Sections were incubated in the absence of the primary antibodies served as negative controls. The tissue sections were washed 4 times with PBS-Triton X-100. The secondary antibodies were anti-mouse Alexa Fluor-fluorescein isothiocyanate (FITC)-conjugated IgM (green fluorescence) and anti-rabbit Alexa Fluor-Cy3-conjugated IgG (red fluorescence; Beijing Zhongshan Goldenbridge Biotechnology), and were incubated for 60 min at 37°C. The tissue sections were washed 3 times with PBS, and the nuclei were stained for 2 min with 4′,6-diamidino-2-phenylindole (DAPI, 1:2,000 dilution), purchased from Sigma-Aldrich (Santa Clara, CA, USA). Following additional washes, images of the tissues were captured by confocal laser scanning microscopy (MTC-600; Bio-Rad, Hercules, CA, USA).

### Western blotting

At each time point for each group, five samples were obtained, with 100 mg per sample. Each solid tissue sample was broken up using ultrasonication, then 0.5–1 ml pyrolysis liquid (Nanjing KeyGEN Biotech, Nanjing, China) was added, mixed thoroughly and span at 14,000 g at 4°C for 30 min. The top layer was then transferred into new tubes and the mixture was centrifuged at 14,000 × g, at 4°C for 10 min and the supernatant was carefully discarded. A mixture of 200 μl buffer and 2 μl protease inhibitors (Nanjing KeyGEN Biotech) were added and centrifuged at 14,000 g for 10min at 4°C. The supernatants were then transferred to new tubes and stored at −80°C. Determination of the protein concentrations was performed using the bicinchoninic acid (Nanjing KeyGen Biotech) method. Subsequently, equal amounts 40 μg) of protein extract were mixed in 1X Laemmli buffer (Nanjing KeyGEN Biotech)., boiled for 5 min, and electrophoresed on precast 7.5% sodium dodecyl sulfate-polyacrylamide gels (80 V for 120 min). The proteins were then electrophoretically transferred onto polyvinylidene difluoride membranes (100 V for 90 min; EMD Millipore, Bedford, MA, USA). The membranes were incubated for 1 h in normal donkey serum (1:10) to block nonspecific binding, and then incubated overnight at 4°C with primary antibodies targeting OLIG1 (1:500 dilution), myelin basic protein (MBP; rabbit monoclonal antibody at 1:1,000 dilution; Abcam), and β-actin (1:2,000 dilution; Abcam), diluted in PBS-0.02% Tween-20. Samples incubated in this solution without primary antibodies served as the negative controls. After washing 3 times in PBS, the membranes were incubated at room temperature for 90 min with the HRP-conjugated secondary antibody. The membranes were then washed in PBS and impregnated with an enhanced chemiluminescence (ECL) substrate (Santa Cruz Biotechnology, Inc., Santa Cruz, CA, USA) to expose the radiographic film. Protein bands were scanned with the ChemiImager 5500 v2.03 software (AlPha InnCh, Miami, FL, USA), integrated digital values were calculated using a computerized image analysis system (Fluor Chen 2.0; Alpha Innotech, San Jose, CA, USA) and normalized to the value of β-actin.

### Reverse transcription-quantitative polymerase chain reaction (RT-qPCR)

The RNAiso Plus kit, manufactured by Takara Bio, Inc. (Dalian, China), was used to extract total RNA. Briefly, 1 ml Trizol reagent (Takara Bio, Inc.) was added to 1–2 g fresh material, mixed thoroughly, cooled on ice for 5 min and centrifuged at 12,000 × g,4°C for 10 min. The supernatants were then transferred to new tubes, 400 μl phenol/chloroform (1:1v) was added and the mixture was vortexed for 15 sec. The samples were then centrifuged at 12,000 × g,4°C for 10 min and the top (aqueous) layer was transferred into new tubes. Isopropanol (1:1v) was added to each tube and samples were centrifuged at 12,000 × g for 10 min at 4°C. The supernatant was discarded and the pellet washed with 1 ml 75% ethanol. Samples were centrifuged again at 7,500 × g for 5 min. Residual ethanol was removed with a pipet. Samples were air-dried for 4 min and the RNA was then redissolved in 30 μl DEPC (diethyl pyrocarboanate, Takara Bio, Inc.). cDNA was synthesized by reverse transcription using the PrimeScript RT reagent kit (Takara Bio, Inc.), and then amplified with a LightCycler real-time PC amplifier (Roche Diagnostics, Mannheim, Germany). The sequences of primers, provided by Takara Bio, Inc., were the following: OLIG1 forward (F), 5′-CCA CAG CAA GGC AGC TGA AG-3′ and reverse (R), 5′-TGC TAA CGC TAA TCA CAA GCC AAG-3′; β-actin F, 5′-GGA GAT TAC TGC CCT GGC TCC TA-3′ and R, 5′-GAC TCA TCG TAC TCC TGC TTG CTG-3′. Reactions were carried out in a total volume of 20 μl, and the amplification conditions were: 95°C for 15 sec and 60°C for 1 min, for a total of 45 cycles. The relative expression level of the *OLIG1* mRNA was calculated with the ΔΔCt method.

### Statistical analysis

Data were summarized as mean ± standard deviation (SD). Statistical analysis was performed by two-way analysis of variance (ANOVA), followed by multiple comparison least significant difference (LSD) tests, using theSPSS 18.0 software (SPSS Inc., Chicago, IL, USA). A difference was considered to be statistically significant at a value of P<0.05.

## Results

### Morphological changes in the brain tissues

**Figure 1:**
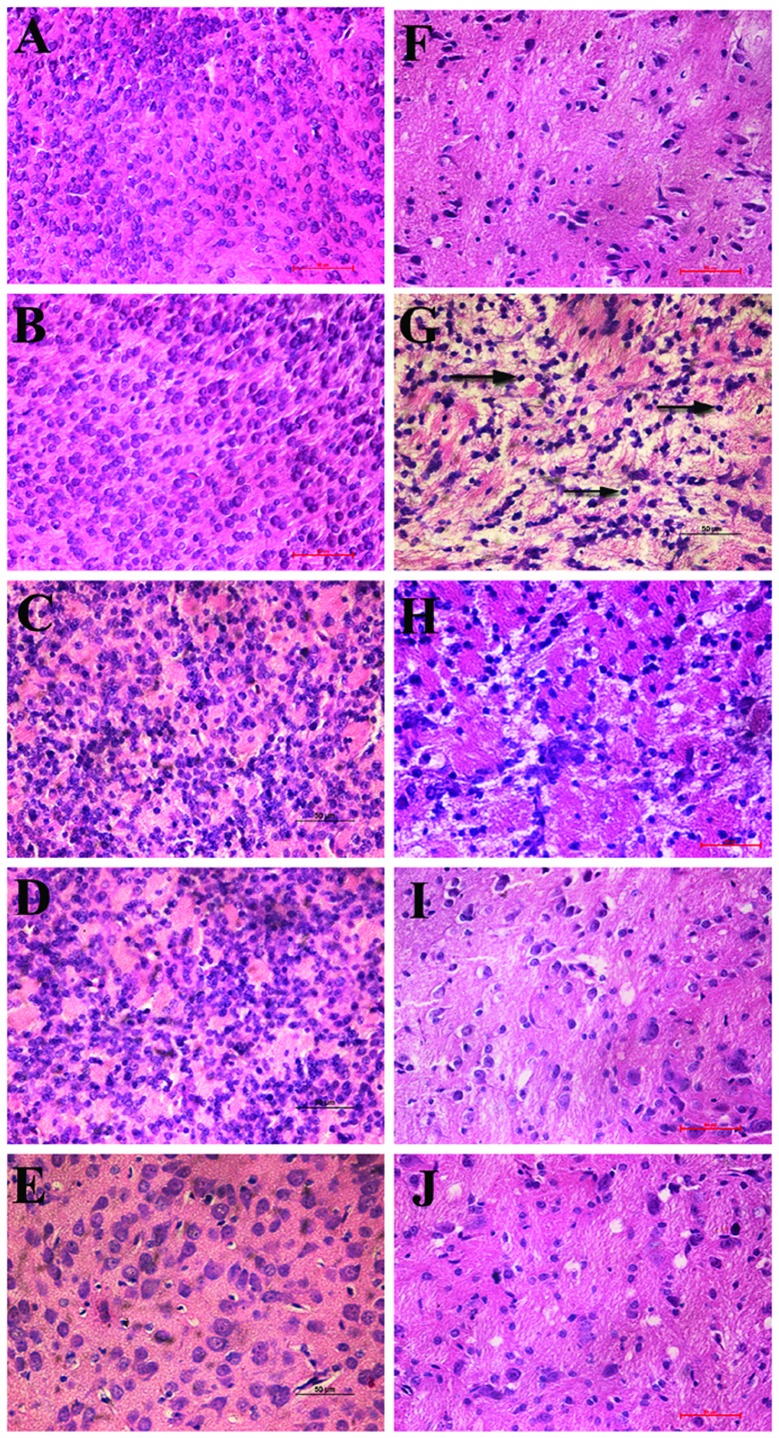
Malacotic periventricular brain tissues caused by hypoxia-ischemia (HI), as observed with hematoxylin and eosin (H&E) staining (magnification, ×400). Brain tissues of the normoxia group exhibited a normal morphology, and uniform, intensive cell distribution at all time-points (A–E). In the HI group, karyopyknosis was observed in brain tissues, with necrosis of a few cells at day 1 (F). At day 3, the focus of malacia began to appear in the alba, and necrosis was observed in a high number of cells, along with a cribriform change (G). At day 7, the number of cystic necroses was reduced compared to day 3, when the necroses first appeared (H). At days 14 and 21 (I,J), the number of cystic necroses was smaller, and cribriform necroses gradually disappeared or were transformed into areas of cluster or punctiform necroses, although brain tissues still had a porous, malacotic structure.

At days 1, 3, 7, 14 and 21, brain tissues in the normoxia group showed a normal morphology and structure; no significant differences were observed among the different time-points (Fig. 1A–E). In the HI group, karyopyknosis and apoptosis were observed in only a few cells at day 1 (Fig. 1F), while at day 3, necrosis following karyopyknosis was observed in numerous cells (indicated by →), and brain tissues had a porous, malacotic structure, in which numerous areas of cystic necrosis vacuoles were observed (Fig. 1G). At day 7, the cribriform structure disappeared, and the number of vacuoles was significantly reduced (Fig. 1H). At day 14, there were progressively increasing numbers of normal cells and much fewer vacuoles (Fig. 1I); at day 21, the total size and number of vacuoles were both significantly decreased, but brain tissues still had a porous, irregular structure (Fig. 1J).

### Ultrastructural changes in the brain tissues

#### HI disrupts the development of OLs

**Figure 2:**
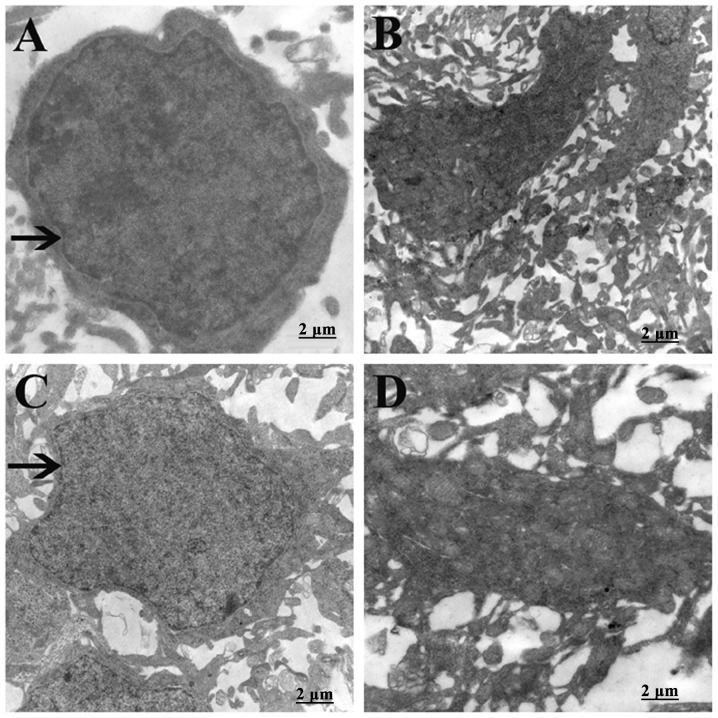
Observation of the effects of hypoxia-ischemia (HI) on oligodendrocytes (OLs) by transmission electron microscopy (magnification, ×25,000). (A and C) In the normoxia group, the morphology and structure of OLs is visible, with a large, full nucleus, and a complete nuclear membrane, indicated by arrows. (B) In the HI group, the cell morphology and structure is highly irregular, with cell atrophy observed, altered nuclear structure, and cell necrosis at day 3. (D) By day 21, the OLs have obtained an irregular structure and show serious cell degeneration and necrosis.

Evaluation of OLs under the electron microscope showed that in the normoxia group, OLs demonstrate a regular morphology and structure, a complete cell membrane structure, a visible nuclear membrane, and a large and visible nucleus, containing uniformly distributed particles of chromatin (Fig. 2A and C). In the HI group, OLs showed a highly irregular morphology, an invisible, damaged nuclear membrane and a vague nucleus, and necrosis was observed in a high number of cells at day 3 (Fig. 2B). At day 21, the nuclear structure had vanished, karyolysis was observed in a few cells, and necrosis of OLs was also observed (Fig. 2D).

#### HI disrupts the maturation of myelin

**Figure 3:**
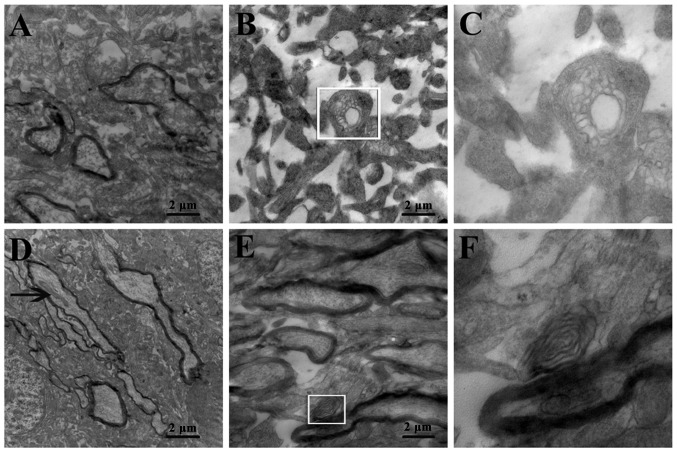
Observation of the effects of hypoxia-ischemia (HI) on the ultrastructure of myelin under the transmission electron microscope (magnification, ×25,000). (A) In the normoxia group, myelin was well-developed, with a compact structure at day 3. (B and C) In the HI group, a large number of small vacuoles was observed in the myelin at day 3 and at day 21. (D) At day 21 in the normoxia group, axons enclosed in myelin were clearly visible (indicated by the black arrows) and the layers exhibited a highly organized appearance. (E and F) Myelin exhibited a loose structure and an increased inter-layer gap with visible stratification, which indicated a disrupted development in the HI group. C and F are the enlarged images of the white boxes shown in B and E, respectively.

In the normoxia group, myelin had a visible morphology and a compact inter-layer structure, in which developing axons were observed (arrows in Fig. 3A and D). In the HI group at day 3, there were numerous small vacuoles formed in the myelin, with a cribriform change, and the development of myelin was clearly altered (Fig. 3B and C). At day 21, an obvious abnormality was still detectable in the structure of myelin, the inter-layer structure was porous, and there was visible stratification in certain layers (Fig. 3E and F).

### Measurement of OLIG1 protein expression in brain tissues by immunohistochemistry

**Figure 4:**
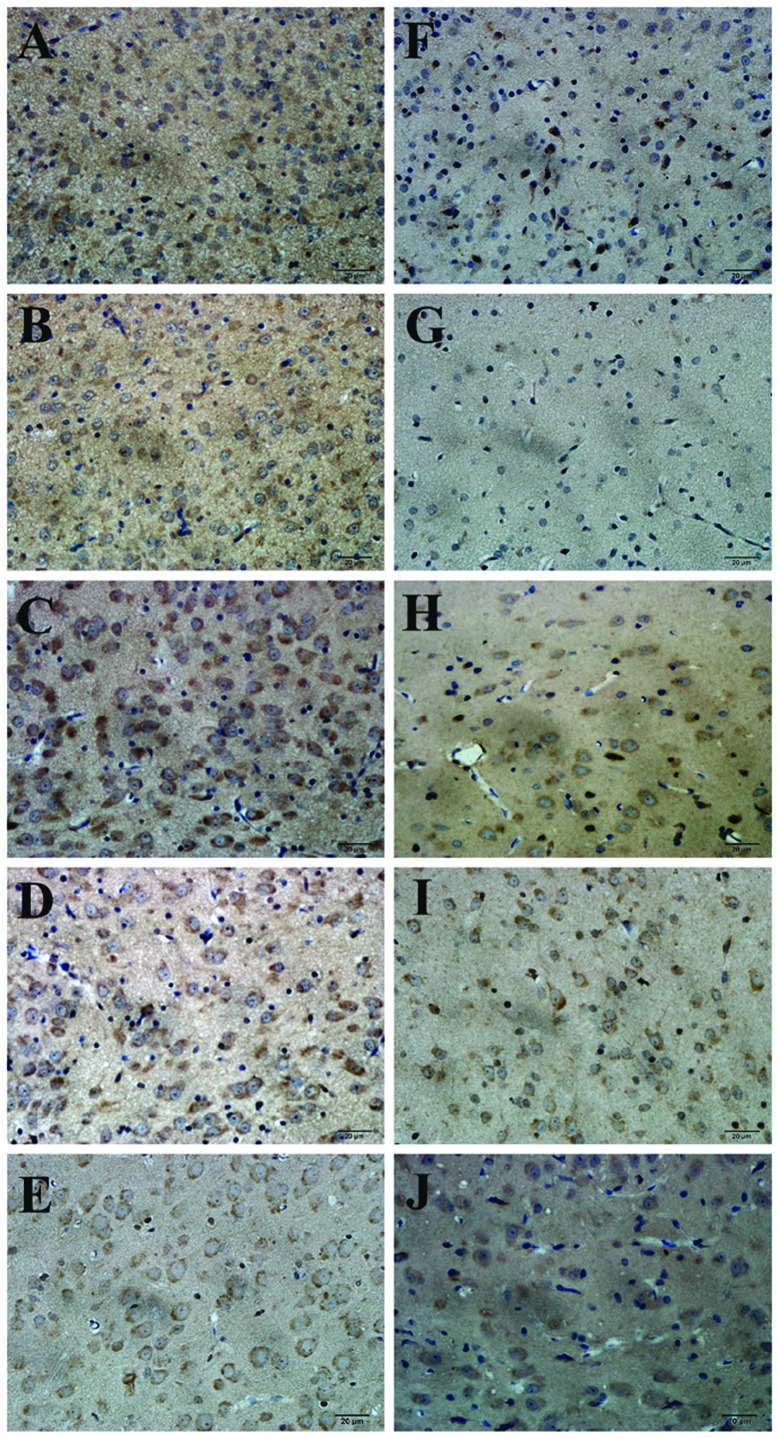
OLIG1 protein expression in brain tissues, as measured by immunohistochemistry (magnification, ×500). At days 1, 3, 7, 14 and 21, the cytoplasm was stained brown (indicating that numerous cells were expressing the OLIG1 protein) in the brain tissues of the normoxia group (A–E). In the hypoxia-ischemia group, only a sparse distribution of cells expressing OLIG1 was observed, with numerous nuclei stained brown at day 1 (F). At day 3 (G), there were substantially fewer positively-stained cells, and at days 7, 14 and 21 (H–J) the positively-stained cells were more extensively distributed, although there remained a high number of yellow-stained nuclei.

At days 1, 3, 7, 14 and 21, a high numbers of cells with their cytoplasm stained brown was observed in brain tissues of the normoxia group, which indicated that numerous cells were expressing the OLIG1 protein (Fig. 4A–E). Compared to the normoxia group, a decreased number of cells with brown staining in the cytoplasm was observed at all time-points in the HI group (Fig. 4F–J), while a high number of nuclei stained brown was observed. These data indicate that the expression of OLIG1 was partially transferred to the nucleus following the HI-induced brain tissue injury.

### Effects of HI on OLIG1 localization in the brain tissues

**Figure 5:**
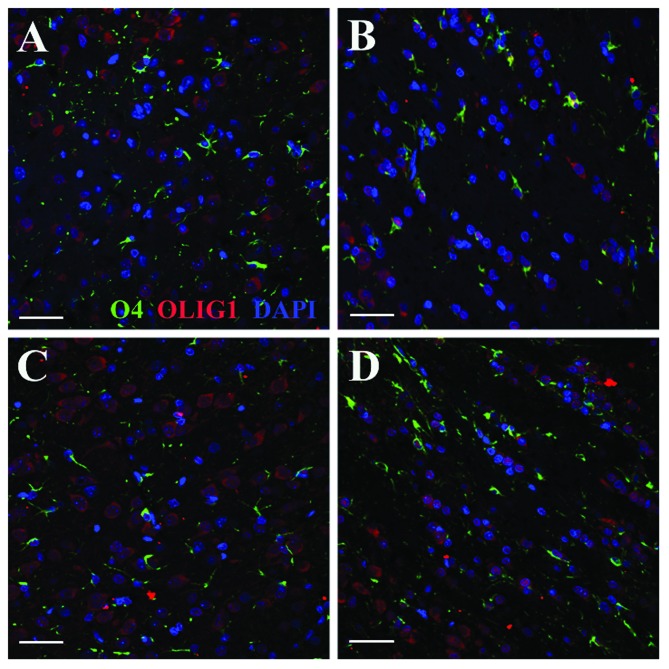
Changes in the cellular localization of OLIG1 in brain tissues following hypoxia-ischemia (HI) (magnification, ×600). (A) Co-expression of O4 and OLIG1 in the cytoplasm without any sign of nuclear translocation was observed in the normoxia group. (B) The expression levels of OLIG1 were reduced compared with those in the normoxia group, and the OLIG1 staining observed was nuclear rather than cytoplasmic in the HI group. (C) At day 21 in the normoxia group, co-expression of O4 and OLIG1 in the cytoplasm was observed. (D) There was reduced expression of OLIG1 in the cell nuclei at day 21 in the HI group compared with the normoxia group, however the expression increased in the cell nuclei following (D) HI at day 21 compared with (C) day 7. Despite the late increase at day 21 in the HI group compared with day 7, the OLIG1 expression levels were still lower compared with those of the normoxia group.

Immunofluorescent double staining revealed co-expression of OLIG1 and O4 (important proteins in mature OPCs) in the cytoplasm of the same cells (Fig. 5A and C); expression of OLIG1 was observed in the cell nuclei of alba cells following HI, but it was reduced compared with that in the normoxia groups (Fig. 5B and D). In the normoxia group, OLIG1 was consistently expressed in the cytoplasm, with no nuclear translocation observed (Fig. 5A and C). These results indicate that OLIG1 is a protein normally expressed in the cytoplasm of healthy OPCs after birth, while under hypoxic and ischemic conditions, it translocates to the nucleus.

### Effects of HI on the protein expression of OLIG1 and MBP in brain tissues

**Figure 6:**
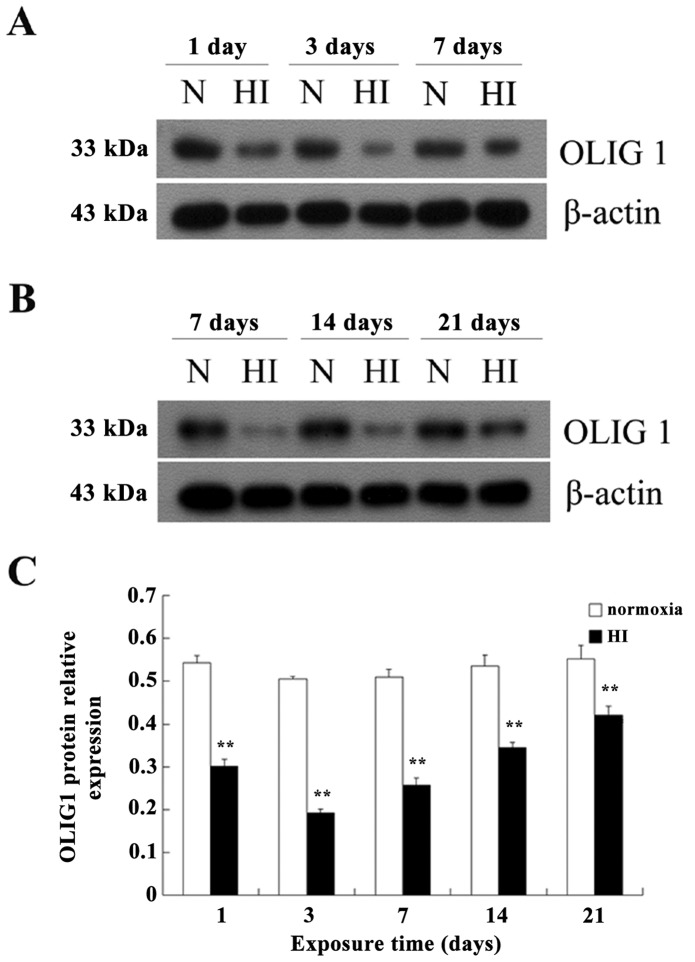

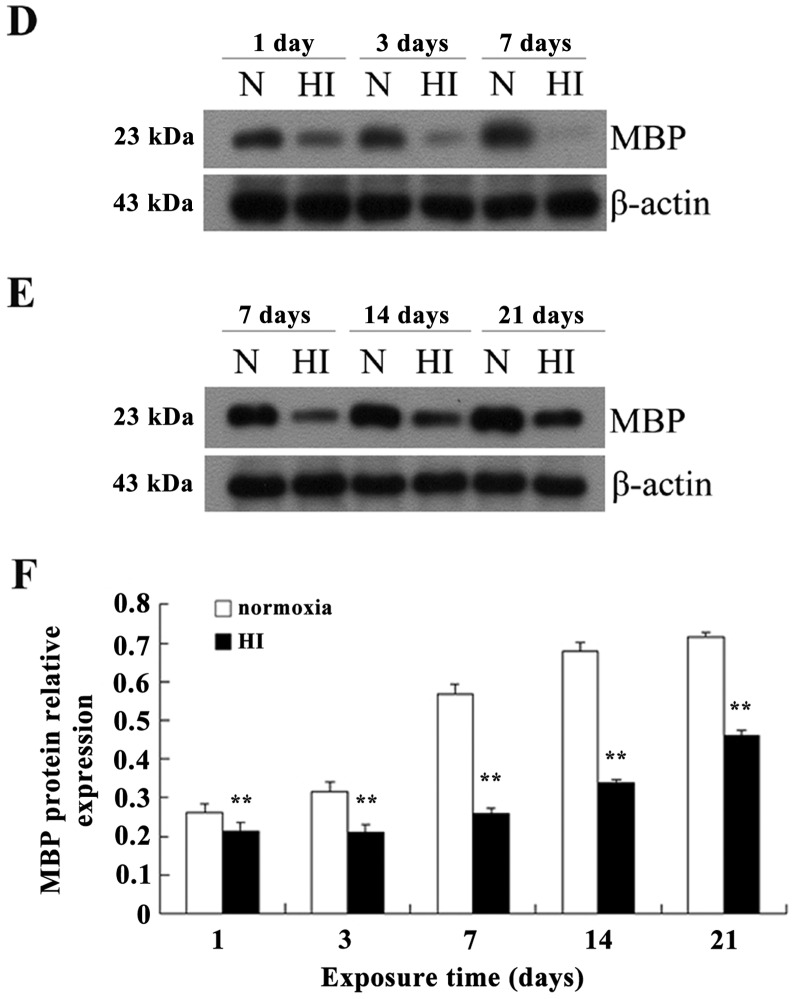
Decreased expression of OLIG1 and myelin basic protein (MBP) protein in brain tissues following hypoxia-ischemia (HI). In the normoxia (N)group, the expression level of OLIG1 in brain tissues is not significantly different (P>0.05) at different time-points (C). In the HI group, the lowest expression level of OLIG1 is observed at day 3 (A). The expression level of the MBP protein in the normoxia group shows a progressively increasing (P<0.01) trend (C). At 7 days of HI exposure, the MBP expression dramatically declines, and is virtually undetectable (P<0.01) by western blotting (D and E). Increased expression of the OLIG1 and MBP proteins is observed in newborn rats at days 14 and 21. In the HI group, despite this increase, the levels of the OLIG1 and MBP proteins (P<0.01) do not reach the expression levels observed in the normoxia group (B–C and E–F). The relative expression level of OLIG1 and MBP proteins (P<0.01) in the brain tissue was calculated by the mean value of the relative optical densities (C and F). Means ± standard deviation (SD) values are shown. ^**^P<0.01 vs. the normoxia group. All experiments were performed independently 4 times.

**Figure 7:**
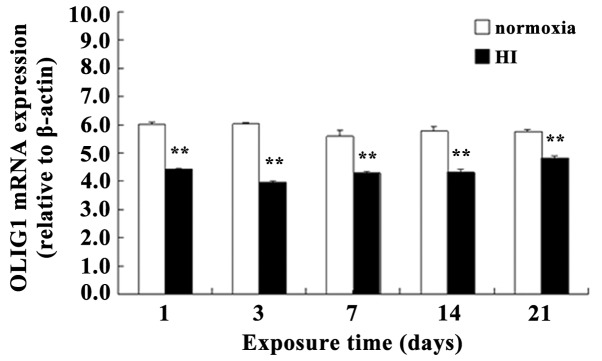
Effects of hypoxia-ischemia (HI) on the *OLIG1* mRNA level in brain tissues. Reverse transcription-quantitative polymerase chain reaction (RT-qPCR) results show no significant difference in the expression level of *OLIG1* in the normoxia group at any time-point (P>0.05), while a marked decrease in the HI group is observed (P<0.01). In the HI group, the expression level of *OLIG1* is the lowest at day 3 and increases at day 21 (P<0.01), but no significant differences are observed among days 3, 14 and 21 (P>0.01). The expression level of *OLIG1* at day 21 is significantly lower than that in the normoxia group (P<0.01). Means ± standard deviation values are shown. ^**^P<0.01 vs. normoxia group. All experiments were performed independently 4 times.

Western blot analysis showed that the protein expression levels of OLIG1 and MBP in the HI group were reduced compared to the normoxia group. No significant difference (P>0.05) in the expression level of OLIG1 was found in the normoxia group at any time-point (Fig. 6C). In the HI group, the expression level of OLIG1 in brain tissue gradually decreased at days 1 and 3, but then increased at days 7, 14 and 21 (Fig. 6B). In the normoxia group, the expression level of MBP progressively and significantly (P<0.01) increased (Fig. 7F). In the HI group, the expression level of MBP reached a peak at day 7 (Fig. 6D) (P<0.01), and then increased at days 14 and 21 (Fig. 6E) (P<0.01). Despite the late increase, the MBP and OLIG1 expression levels were still lower (P<0.01) than those of the normoxia group (Fig. 6C and F).

### Decreased expression of OLIG1 mRNA following HI-induced brain injury

At all time-points, there was no significant difference in the expression level of the *OLIG1* mRNA in the normoxia group (P>0.05), but there was a significant decrease in the HI group as compared to the normoxia group (P<0.01). In the HI group, this decrease began at day 1 (P<0.01), and reached a peak at day 3 (P<0.01); then, a slight increase was observed at days 7 and 14, but the difference between days 3 and 7 or day 14 was not statistically significant (P>0.05). The increase in the level of the *OLIG1* mRNA observed at day 21 was statistically significant compared to the levels at days 1 and 3 (P<0.01), but was not significant when compared to the levels at days 7 and 14 (P>0.05).

## Discussion

PVL is the most serious sign of WMD in premature infants. Its incidence in this group is 8–26% in the developing countries, and 5–17% in premature infants weighing <1,500 g, with >66% of these infants developing cerebral paralysis (13). In developed countries however, only 10% of PVL patients develop cerebral paralysis, and 25–50% of these patients suffer from complicated cognitive disorders, behavioral deficiencies, and mild dyskinesia (14). At present, PVL is the most common disease seriously affecting the quality of life and long-time survival in premature infants. The current hypotheses on the mechanisms underlying PVL include the abnormal secretion of inflammatory factors and the deleterious effects of oxyradicals (15,16). However, most studies have rarely focused on HI as the molecular mechanism of PVL. A study conducted by Craig *et al* (17), showed that newborn rats aged 2–5 days show immature nerve development equivalent to that of a human fetus at a gestation age of 28–32 weeks, which is consistent with the time period when alba injury occurs in humans during the perinatal period. The PVL model we used in our study is very similar to PVL in premature infants with respect to the pathology and clinical manifestations, and we therefore investigated the effects of OLIG1 on PVL-related WMD in a 3-day newborn rat model of HI-induced PVL.

PVL is frequently observed during the peak of the myelin period (18), and when there is an abundance of advanced-stage OPCs (accounting for ~90% of OLs) in newborn rat brain tissues. The OPCs will further develop and form axon-enclosing myelin, and are highly sensitive to HI (19). The stimulation of OPCs by HI at 2–5 days after birth disrupts myelin and causes PVL (20). OLs are currently considered the major cell target of PVL (21), and the deposition of myelin is not complete in the above-mentioned period; thus the selective vulnerability of OPCs is the main contributor to cerebral paralysis, which is a severe *sequela* of PVL (22). It was reported that HI not only disrupts the development and maturation of OLs (23), but also results in changes in OLIG1 expression (24,25). French *et al* (24) showed that oxidative stress following HI reduces the expression of OLIG1 and hinders the differentiation and maturation of nerve stem cells to OLs. Furthermore, Tanaka *et al* (25) found that the expression of OLIG1 and other transcription factors in adult monkey brain tissue is decreased, followed by an increase during the process of ischemic brain injury. This study showed that the expression of OLIG1 in newborn rat brain tissues is decreased at the early stage, but then increases to levels lower than the normal at the late stage following HI exposure. Therefore, the dynamic changes in OLIG1 expression in the presence of HI-induced WMD and the role of OLIG1 in promoting the repair and regeneration of OLs and myelin following PVL brain injury need both to be confirmed by future studies.

In this study, we observed porous, malacotic brain tissues, karyolysis followed by OL necrosis, formation of small vacuoles, and a loosened inter-layer myelin structure following HI exposure. Furthermore, we observed a significant decrease in the expression of OLIG1 and MBP after HI, which is consistent with OL and myelin injury. These findings reveal that the abnormal protein expression of OLIG1 and MBP following PVL brain injury caused by HI plays an essential role in disrupting the development and maturation of OLs and myelin.

The current study showed that the downregulation of OLIG1 during the process of HI-induced PVL brain injury may affect the maturation and development of myelin. OLIG1 is an important indicator of OPCs (9,26), and it can stimulate their development to mature OLs (27); myelin is then formed by the development of OLs (17). OPCs are highly sensitive to the effects of HI (28,29), and we found nearly parallel changes in OLIG1 and MBP expression levels, which is consistent with the developmental disorders affecting OLs and myelin. Therefore, downregulation of the *OLIG1* gene in hypoxic-ischemic brain tissues will lead to a decrease in the expression of certain proteins that are required to form myelin (e.g., MBP), and thus delay the development of myelin.

Our findings demonstrate that brain tissues have a certain ability of self-repair following HI; however, myelin cannot completely be repaired. The expression level of OLIG1 in brain tissues showed a decreasing trend at the early stage, and an increasing trend at the late stage following HI, but failed to reach the levels observed in the normoxia group. In addition, a change in the localization of OLIG1 was also observed; i.e., similar to other studies (26,30), OLIG1 was expressed in the cytoplasm of OLs in normoxic brain tissues after birth. OLIG1 was also highly expressed in the nuclei of OPCs after the initiation of developmental disorders that were secondary to myelin injury caused by HI (31). Thus, OLIG1 promoted the development of immature OPCs to mature OLs, and eventually contributed to the migration of mature OLs to newly formed myelin. This change in the nuclear translocation of OLIG1 may enhance the regeneration of myelin. Following HI, the protein expression level of MBP was increased at the late compared to the early stage, which is a pattern very similar to that of OLIG1; this suggests that OLIG1 has a certain promoting role in the repair of myelin. However, despite this repair effect of OLIG1, myelin was not fully repaired to a normal level.

The earliest manifestation of HI-induced PVL is degeneration and necrosis in a few cells, with brain tissues maintaining a visible structure, and intensively arranged nerve fibers. This is followed by liquefactive necrosis in a higher number of cells, a porous structure of brain tissues with even visible small vacuoles left by liquefactive necrosis, and irregularly arranged nerve fibers. The vacuoles left by cell necrosis of the brain tissue eventually become reduced in number and size with the progressive development of brain tissue. At 21 days following HI, TEM observations revealed serious damage in a considerable number of OLs and myelin in brain tissues of the HI group, as compared to the normoxia group. The downregulation of OLIG1 following hypoxic-ischemic WMD can also result in the reduced expression of myelin-related proteins such as MBP, and thus cause delayed myelination and development of PVL. OLIG1 can promote myelination by stimulating nuclear transcription, but this effect is insufficient for the complete repair of the injured myelin. Additional studies need to be conducted to determine whether *OLIG1* gene therapy may efficiently prevent and treat PVL in premature infants with PVL-related WMD.
